# Influence of Drying Methods on Redispersibility and Dissolution of Canagliflozin Nanocrystals: A Comparative Approach

**DOI:** 10.3390/ph19020240

**Published:** 2026-01-29

**Authors:** Yagmur Pirincci Tok, Burcu Demiralp, Sevgi Güngör, Ali Osman Sarikaya, Emre Erol Aldeniz, Udaya Kumar Dude, Yildiz Ozsoy

**Affiliations:** 1 Department of Pharmaceutical Technology, Faculty of Pharmacy, İstanbul Health and Technology University, İstanbul 34275, Türkiye; yagmur.tok@istun.edu.tr; 2 Department of Pharmaceutical Technology, Faculty of Pharmacy, İstanbul University, İstanbul 34126, Türkiye; bmesut@istanbul.edu.tr (B.D.); sgungor@istanbul.edu.tr (S.G.); 3 Research and Development Center, Abdi İbrahim Pharmaceutics, Istanbul 34538, Türkiye

**Keywords:** nanocrystal, solidification, spray-drying, fluidized bed granulation, improved dissolution rate

## Abstract

**Background/Objectives**: Canagliflozin (CFZ) is the first sodium glucose co-transporter 2 (SGLT-2) inhibitor and is characterized by poor water solubility and permeability, resulting in low oral bioavailability. In this study, a CFZ nanosuspension (CFZ-NS) was converted into a solid form to improve the physical stability of CFZ nanocrystals (CFZ-NCs) and to enable formulation as a tablet dosage form. **Methods**: To achieve adequate redispersibility of dried CFZ-NCs, fluid bed granulation and spray-drying methods were employed, and the effects of critical process parameters were investigated. The stability of spray-dried nanocrystal tablets (NCs-SD-TAB) was evaluated over a three-month period under storage conditions of 25 ± 2 °C with 60 ± 5% relative humidity (RH) and 40 ± 2 °C with 75 ± 5% RH. **Results**: The highest redispersibility index (94%) was obtained using the spray-drying method. Tablets prepared with spray-dried NCs-SD-TAB exhibited a significantly higher in vitro dissolution rate under non-sink conditions compared with control tablets prepared using unprocessed CFZ with the same excipients, as well as the marketed product. NCs-SD-TAB showed an approximately three-fold increase in drug release at 15 min in 0.1 N HCl, with a pH 4.5 acetate buffer and pH 6.8 phosphate buffer, which simulate gastrointestinal pH conditions, relative to the marketed product. **Conclusions**: Overall, these results indicate that nanocrystal technology represents a promising approach for CFZ as an improved oral drug-delivery system, primarily due to its solubility enhancement capabilities.

## 1. Introduction

The International Diabetes Federation estimates that 537 million people aged 20–79 years had diabetes worldwide in 2021, and this figure may rise to 783 million by 2045 [1]. Although Diabetes Mellitus (DM) has been recognized as a clinical problem for more than two centuries, its treatment and management remain challenging [2], and the disease is considered the sixth leading cause of mortality worldwide [3]. In recent years, nanocrystal technology has attracted increasing interest in anti-diabetic drug delivery due to its potential to enhance dissolution-limited oral absorption. While many studies report improved in vitro dissolution rates, achieving consistent bioavailability and therapeutic benefits in humans depends on the specific drug and formulation [4]. Nanosuspension-based systems can also enable modulation of drug release behavior, potentially yielding more stable plasma drug levels and improved glycemic control, which may reduce the risk of acute hypoglycemia and long-term diabetic complications [5].

Canagliflozin (CFZ), the first member of the SGLT2 inhibitors, lowers plasma glucose levels by increasing urinary glucose excretion in patients with type 2 DM, causing mild osmotic diuresis and a net calorie loss [6]. Beyond lowering glucose, they reduce glycated hemoglobin (HbA1c) levels and are associated with improved renal outcomes and reduced cardiovascular events through multiple mechanisms, including renoprotection, the attenuation of glomerular hyperfiltration and albuminuria, enhanced sodium excretion, and a concomitant reduction in blood pressure [7,8]. On the other hand, CFZ is practically insoluble in aqueous media over a wide pH range (pH 1.1–12.9) [9], and its absolute oral bioavailability is approximately 65%, indicating poor to moderate permeability. Accordingly, the applicants Mitsubishi Tanabe Pharma and Johnson and Johnson have classified CFZ as a Biopharmaceutical Classification System (BCS) Class IV drug [10]. Low oral bioavailability therefore remains a major limitation for certain contemporary diabetes treatments [11]. It is well established that the oral absorption of active pharmaceutical ingredients with low water solubility can be highly variable, potentially leading to inconsistent systemic exposure. However, many anti-diabetic drugs that are currently available on the market are adequately soluble and can be formulated successfully without the need for specialized solubilization strategies. In contrast, for poorly water-soluble anti-diabetic compounds, dissolution-limited absorption can be a key factor affecting oral bioavailability and therapeutic performance [12].

Nanocrystal (NC) technology is one of the most widely explored approaches among nanotechnology-based drug delivery systems, with more than 20 NC drug products currently available on the pharmaceutical market [13]. The widespread adoption of NCs by both the pharmaceutical industry and researchers is primarily attributed to their submicron particle size (<1000 nm). They are especially commonly produced by the wet grinding technique, yielding particles in the range of 200–400 nm dispersed in aqueous media with stabilizers; such systems are therefore often referred to as nanosuspensions. By definition, nanocrystal systems are carrier-free formulations composed of the active pharmaceutical ingredient stabilized by small amounts of surface-active agents, in contrast to most nanocarrier-based formulations, which incorporate substantial amounts of excipients. This characteristic enables a theoretical drug loading of up to 100% while also allowing a simplified formulation design and greater flexibility for scaling up [14]. Moreover, owing to their carrier-free nature and reduced dependence on excipients, nanocrystals generally require fewer stabilizers and may exhibit lower toxicity than carrier-based systems such as nanoparticles and liposomes [15]. Particle size reduction increases the surface area and surface free energy, thereby enhancing saturation solubility [16], dissolution rate [17], enhanced transport across the intestinal membrane [16,18], and ultimately oral bioavailability [19,20]. Nevertheless, drug nanosuspensions face significant challenges related to their physical stability. As colloidal dispersions, they exhibit a strong tendency to aggregate in order to minimize their surface energy and may undergo time-dependent, solution-mediated recrystallization phenomena whereby material redistribution between particles can occur [21]. Addressing these physical stability issues is therefore essential for the successful development of a nanocrystal-based drug delivery system.

Considering the thermodynamic instability of nanosuspensions, the conversion of drug nanosuspensions into a solid state by the drying process represents a valuable approach to achieving optimal stability [22]. Although the drying process is a critical step in transforming nanosuspensions into solid forms, it may induce particle aggregation or particle growth due to thermal stress and the removal of the liquid phase [23]. Therefore, a thorough understanding of the drying process and critical parameters that strongly influence the redispersibility of dried powders is essential, along with the careful selection of an appropriate drying method [23,24]. In the drying process, various techniques are employed, including freeze drying, spray-drying, vacuum drying, pelletisation, granulation/coating in a fluidized bed dryer, drum drying, and electro-spraying [25]. In this study, fluidized bed granulation and spray-drying were selected, as the former is widely preferred by the pharmaceutical industry, and the latter is extensively used for the drying of nanosuspensions.

The drying of nanosuspensions not only ensures optimum stability but also enables their conversion into solid dosage forms, such as capsules and tablets, and is suitable for oral administration [26]. In the present study, CFZ-NS optimized in our previous study [27] was dried using a suitable method and subsequently transformed into a tablet dosage form via the direct compression (DC) method. DC is a preferred technique as it involves a minimum number of processing steps, eliminating the need for intermediate procedures such as granulation and thereby reducing both production costs and time [28]. However, in order to produce tablets with adequate mechanical strength and content uniformity, both active ingredients and excipients must exhibit suitable flowability, compressibility, compactibility, and limited elastic recovery.

This study mainly focuses on the conversion of CFZ-NS into a solid form with desirable redispersibility. To achieve this objective, top spray granulation and spray-drying methods were employed, and the effects of key process parameters on particle size and in vitro drug release behavior were systematically investigated. To facilitate practical application, the dried CFZ-NCs were subsequently formulated into a tablet dosage form using excipients suitable for DC. The resulting tablets were evaluated for weight variation, content uniformity, breaking strength, friability, in vitro disintegration time, and in vitro drug release under non-sink conditions. In addition, stability studies were conducted for 3 months under controlled conditions of 25 ± 2 °C with 60 ± 5% RH and 40 ± 2 °C with 75 ± 5% RH. Overall, this work aims to develop nanocrystal-based tablet formulations with enhanced dissolution performance, which may ultimately improve patient compliance.

## 2. Results and Discussion

### 2.1. Preparation of CFZ-NS

As described in our previous study [27], CFZ-NS was prepared by wet stirred media milling, yielding a mean particle size of 204.60 ± 5.22 nm (particle size distribution curves are shown in Figure S1 in the Supplementary Materials) with a PDI of 0.147 ± 0.05. In that study, Differential Scanning Calorimetry (DSC) and X-ray powder diffractometry (XRPD) analyses confirmed that the crystallinity and polymorphic form of the prepared nanosuspension were preserved throughout the wet grinding process. The same nanosuspension formulation, prepared following the previously reported method, was used as the starting material for the drying experiments in the present work. The particle size of the CFZ-NS employed for each drying experiment is summarized in Table 1.

### 2.2. HPLC Analysis

The method exhibited linearity over the concentration range of 12–200 µg/mL for the investigated media, with a coefficient of determination (R^2^) greater than 0.999. The limits of detection (LOD) and quantification (LOQ) were 3.5 µg/mL and 11.5 µg/mL, respectively. In addition, the selectivity of the method in the presence of excipients was confirmed.

### 2.3. Solidification of CFZ-NCs by Fluidized Bed Granulation and Spray-Drying Methods

In the present study, a series of experimental runs (i.e., NCs-FBG and NCs-SD) were conducted to identify critical process parameters associated with fluidized bed granulation and spray-drying, and their effects on redispersed particle size are summarized in Table 1. Among the fluidized bed granulation studies, the greatest particle growth was observed in the NCs-FBG2 run, while the most pronounced particle growth during spray-drying occurred in NCs-SD2^a^. In both cases, the relatively high outlet air temperatures associated with elevated inlet air temperature were identified as notable factors. As reported by Laier et al. [29], inlet air temperature is the primary determinant of outlet air temperature in spray-drying, although aspiration rate and feed rate also play important roles. Accordingly, in the NCs-SD2^a^ experiment, reducing the feed rate under high-temperature conditions contributed to particle growth.

When comparing NCs-FBG2 with NCs-FBG1, the influence of carrier material redispersibility became evident. Although identical process parameters were applied, replacing lactose with mannitol resulted in increased particle growth. This behavior can be attributed to the physicochemical properties of mannitol, which is less hygroscopic and more crystalline, leading to a lower capacity to retain the sprayed liquid phase. Such characteristics may promote less uniform binder distribution and increased particle–particle contact during granulation. Consequently, a higher tendency toward agglomeration was observed in the mannitol-rich formulation (NCs-FBG2), whereas lactose-based formulations exhibited comparatively lower agglomeration due to more favorable liquid retention and binder spreading. These findings highlight the sensitivity of nanocrystal-based systems to carrier selection during fluidized bed granulation.

Furthermore, binary studies (i.e., NCs-FBG1/NCs-FBG2 and NCs-FBG3/NCs-FBG4) demonstrated that the redispersibility is strongly influenced by both the carrier type and its particle size, in agreement with the literature [30,31]. In addition, increased moisture content is known to promote particle agglomeration during fluidized bed processing [32]. Although the inlet air temperature applied in NCs-FBG1 was higher than that in NCs-FBG5, more pronounced particle growth was observed for NCs-FBG5. This is most likely attributable to the higher residual moisture content of the NCS-FBG5 sample, which was approximately 2-fold greater than that of NCs-FBG1 (for moisture content results, see Table S1 in Supplementary Materials).

Capillary pressure theory is commonly used to explain agglomeration phenomena in various nanoparticle systems during drying and is equally applicable to nanocrystal formulations. According to this theory, agglomeration arises from capillary forces generated during the solvent evaporation [33]. As the liquid phase evaporates, the curved liquid meniscus between adjacent nanocrystals produces capillary forces that draw particles closer together and promote their coalescence into agglomerates. Higher residual moisture content can further intensify these capillary forces by sustaining liquid bridges between particles for a longer duration, thereby leading to increased agglomeration [34,35].

The highest redispersibility index was observed for NCs-FBG3 among the fluidized bed granulation experiments and for NCs-SD6^c^ in the spray-drying studies. In NCs-FBG3, reducing the pump speed by half lowered the relative humidity within the fluidized bed, thereby limiting nanocrystal agglomeration to some extent and resulting in an RDI of approximately 75% [31,36]. A similar trend was reported by Figueroa et al. [32], who observed a reduction in particle size when the spray rate was decreased from 2.5 g/min to 1.8 g/min. Based on the findings of this study, maintaining bed moisture at an appropriate level—by reducing the pump speed or by increasing the amount of carrier material—appears to be critical for controlling the redispersed particle size. However, such approaches may not be practically sustainable, as they tend to prolong process time and/or reduce active ingredient yield, as observed in our study. In the case of NCs-SD5^c^, lowering the inlet air temperature to 70 °C and reducing the feed rate to 10% decreased both the outlet air temperature and the moisture content of the drying air, thereby markedly limiting agglomeration and yielding dried CFZ-NCs with an RDI of 86%. The Peclet number (Pe) is a measure of the relative rates of solvent evaporation and nanoparticle diffusion within a spray-dried droplet [37] and can be used to predict whether nanoparticles can redistribute uniformly inside the droplet before drying is completed. As defined in Equation (1), R^2^/D represents the diffusion time of nanoparticles from the droplet surface to its center, where R is the droplet radius, D is the diffusion coefficient, and τd is the drying time.

Pe = R^2^/τd D (1)

In general, a high Peclet number, as observed in short drying times and rapid drying rates, indicates that nanoparticles have limited time to diffuse toward the droplet center. As a result, particles tend to accumulate at the droplet surface, increasing the likelihood of nanoparticle agglomeration or coagulation. On the other hand, a low Peclet number reflects a slower drying process that allows sufficient time for nanoparticles to diffuse from the surface to the core of the droplets. This promotes a more homogeneous particle distribution within spray-dried powder, thereby reducing agglomeration and enhancing redispersibility [38,39].

In the NCs-SD6^c^ study, the highest yield of redispersible nanocrystals was obtained by maintaining the process parameters of NCs-SD5^c^ and incorporating mannitol into the formulation at a 2:1 ratio. The role of mannitol in enhancing redispersibility can be attributed to the formation of a continuous matrix surrounding the nanocrystals during the spray-drying, which prevents irreversible aggregation. Upon rehydration, mannitol readily dissolves as a hydrophilic excipient, allowing the nanosuspension to be reconstituted [40]. To ensure that dried drug nanocrystals remain as individual nanoscale units without aggregation, it is a common strategy to add matrix-forming excipients—such as low-molecular-weight sugars (e.g., sucrose, trehalose), sugar alcohols (e.g., mannitol), or high-molecular-weight sugar polysaccharides (e.g., maltodextrin)—to the nanosuspension prior to the drying process [23,41]. Similarly, in the NCs-SD4^b^ run, a smaller redispersed particle size was obtained despite a higher outlet air temperature compared with NCs-SD2^a^, most likely due to the presence of mannitol. Moreover, stabilizers in the nanosuspension formulation played a critical role in enhancing the redispersibility of NCs, alongside the effect of mannitol. In particular, the combination of electrostatic and steric (electrosteric) stabilization has been shown to be an effective approach to prevent nanocrystal agglomeration in response to pH variations within the gastrointestinal tract, offering advantages over electrostatic stabilization alone [42].

The present study revealed that the spray-drying produced CFZ-NCs with superior redispersibility compared with fluidized bed granulation. Spray-drying has been widely adopted in the pharmaceutical industry for decades due to its high reproducibility, continuous production, large processing capacity, and fast drying capability [43]. However, scalability remains a challenging issue due to the complex physical transformations that occur during the process.

**Figure 1:**
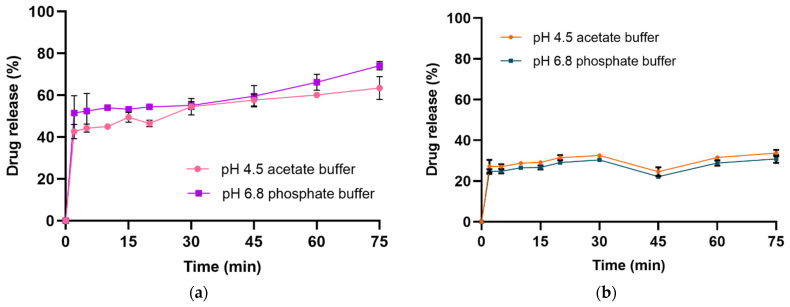
Dissolution profiles of dried CFZ-NCs of NCs-SD6^c^ (**a**) and NCs-FBG3 (**b**) under non-sink conditions.

In vitro drug release studies were conducted on dried CFZ-NCs with the highest redispersibility indices (i.e., NCs-FBG3 and NCs-SD6^c^) to evaluate the extent to which particle size growth affected the dissolution rate of the redispersed nanosuspension (Figure 1). In pH 4.5 and pH 6.8 dissolution media, dried CFZ-NCs obtained from NCs-FBG3 exhibited drug release values of 33.80 ± 1.58% and 30.92 ± 2.00%, respectively, while those obtained from NCs-SD6^c^ exhibited substantially higher release values of 63.43 ± 2.84% and 74.15 ± 5.13%. Consistent with the redispersibility index, the in vitro drug release rate decreased with increasing particle growth. The spray-dried particles displayed a non-monotonic, time-dependent dissolution profile, characterized by an initial rapid release, followed by an apparent plateau and a subsequent increase in release. The second increase observed after approximately 45 min can be attributed to time-dependent wetting and progressive deagglomeration of the porous spray-dried matrix. As the matrix erosion proceeds and porosity increases, drug entrapped within agglomerates becomes increasingly accessible to the dissolution medium, resulting in enhanced drug release.

Across all fluidized bed granulation experiments (NCs-FBG1 to NCs-FBG6), the resulting granules exhibited comparable release profiles, with average release values ranging from 28% to 33% (Figure S2, Supplementary Materials). In accordance with the classical thermodynamic and diffusion-based dissolution principles, as described by the Ostwald–Freundlich and Noyes–Whitney relationships, the reduction in effective surface area associated with particle growth led to a decline in the dissolution rate of dried CFZ-NCs [44]. Under these conditions, the dissolution enhancement afforded by nanocrystal technology was largely negated by particle agglomeration occurring during fluidized bed granulation. Furthermore, it may be argued that the increased adhesion to gastrointestinal cell membranes provided by NC technology is compromised as a consequence of particle growth [45]. This loss of nanoscale characteristics may ultimately limit drug permeation and contribute to variability in oral bioavailability [46].

It is important to note that conducting dissolution tests under non-sink conditions is the preferred approach for evaluating drug nanosuspensions [47,48,49,50], as it allows for greater discrimination between different nano-formulations. In contrast, the FDA-recommended dissolution medium for the marketed product contains 0.75% SLS [51] to ensure sink conditions. At such a high concentration, exceeding the critical micelle concentration, SLS acts as a solubilizing agent, thereby masking differences between dissolution profiles of nanosuspensions and limiting their discriminatory power [31]. Furthermore, dissolution profile analysis under sink conditions provides insight into the in vivo behavior of developed formulations and the progressive release of the drug within the gastrointestinal tract [52]. To achieve this objective, the in vitro drug release rate of the dried CFZ-NCs (i.e., NCS-FBG and NCs-SD) was evaluated in pH 4.5 acetate buffer and pH 6.8 phosphate buffer without adding SLS.

### 2.4. Morphology and Solid-State Evaluation

**Figure 2:**
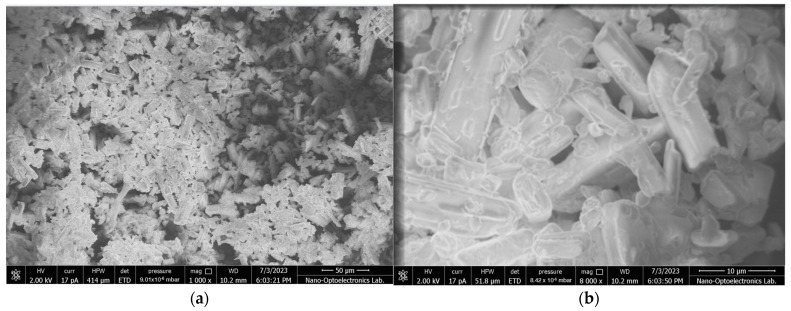

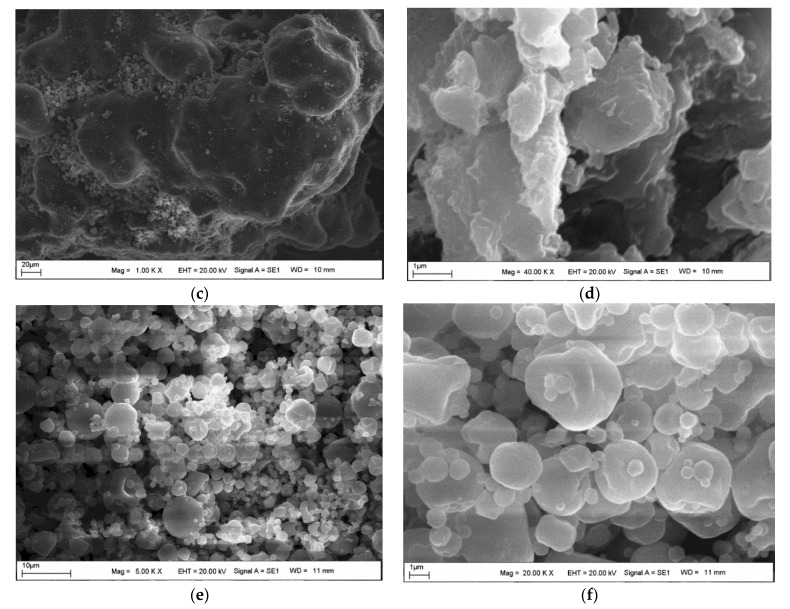
SEM images of unprocessed drug (**a**,**b**), NCs-FBG3 (**c**,**d**), NCs-SD6^c^ (**e**,**f**).

SEM images illustrating surface morphology of unprocessed CFZ and dried CFZ-NCs with the highest redispersibility obtained using both drying methods (i.e., NCs-FBG3 and NCs-SD6^c^) are provided in Figure 2. The SEM image of unprocessed CFZ showed elongated, rhombohedral crystals with heterogeneous shapes and sizes (Figure 2a,b) predominantly forming aggregated clusters. The SEM results indicated that the surface morphology of the dried CFZ-NCs was completely changed compared to the unprocessed drug. As illustrated in Figure 2c,d, the dried CFZ-NCs exhibited a marked tendency toward aggregation, with particles progressively approaching one another and ultimately undergoing partial fusion. This phenomenon can be attributed to the diminution of interparticle boundaries, resulting in a loss of particle distinctness and individuality, accompanied by clear evidence of nanocrystal coalescence [53]. In contrast, SEM images of NCs-SD6^c^ (Figure 2e,f) confirmed that the particles largely retained their individuality and displayed acceptable homogeneity consistent with the result of the redispersibility studies.

**Figure 3:**
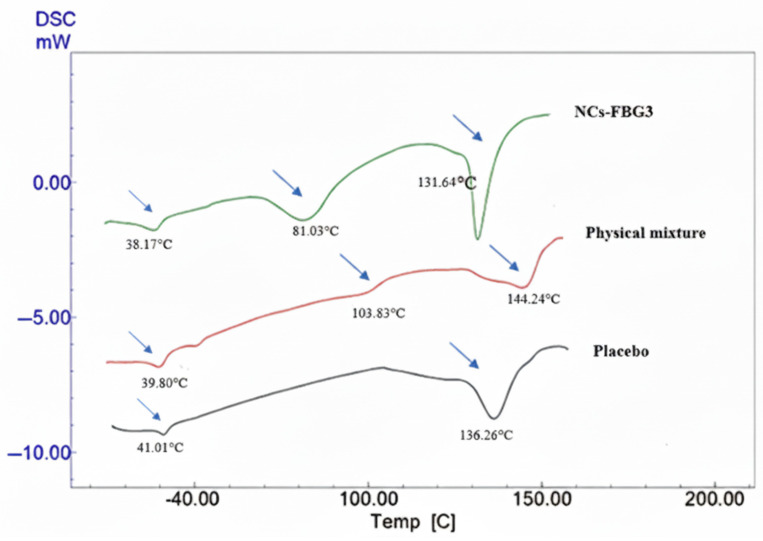
DSC curve of placebo, physical mixture, and fluidized bed-granulated nanocrystals (NCs-FBG3).

In order to investigate the crystalline properties of the dried CFZ-NCs and to compare the effects of the drying methods, DSC and XRD analyses were carried out on dried CFZ-NCs obtained by fluid bed granulation (NCs-FGB3) and spray-drying (NCs-SD6^c^), which exhibited the highest redispersibility. As shown in Figure 3, the placebo formulation of FBG-3 displayed endothermic peaks corresponding to P407 at 41.00 °C and lactose monohydrate at 136.26 °C. In the physical mixture containing CFZ, the endothermic events at 39.80 °C (P407), 103.83 °C (CFZ; for a DSC curve of API, see Figure S3), and 144.24 °C (lactose monohydrate) indicate the presence of individual components without evidence of strong solid-state interactions. Following fluidized bed granulation, the DSC thermogram of dried CFZ-NCs exhibited noticeable shifts in the characteristic thermal transitions of the formulation components. The P407-related endotherm shifted to 38.17 °C, while the CFZ-related peak was observed at 81.03 °C. In addition, the lactose monohydrate endotherm appeared at 131.64 °C. These shifts toward lower temperatures are indicative of reduced crystallite size, increased surface area, and altered thermal behavior arising from nanocrystal formation and intimate mixing with excipients during the granulation process, rather than polymorphic transformation.

**Figure 4:**
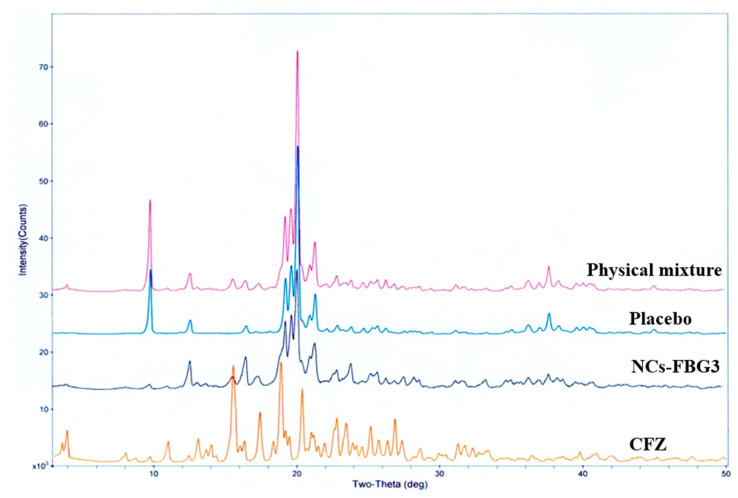
XRPD diffraction patterns of CFZ, placebo physical mixture, and fluidized bed-granulated nanocrystals (NCs-FBG3).

The characteristic lines of CFZ (Figure 4) were also observed both in the physical mixture and in granules (dried NCs-FBG3), albeit with a noticeable reduction in intensity, as expected due to the reduced particle size and lower drug content.

**Figure 5:**
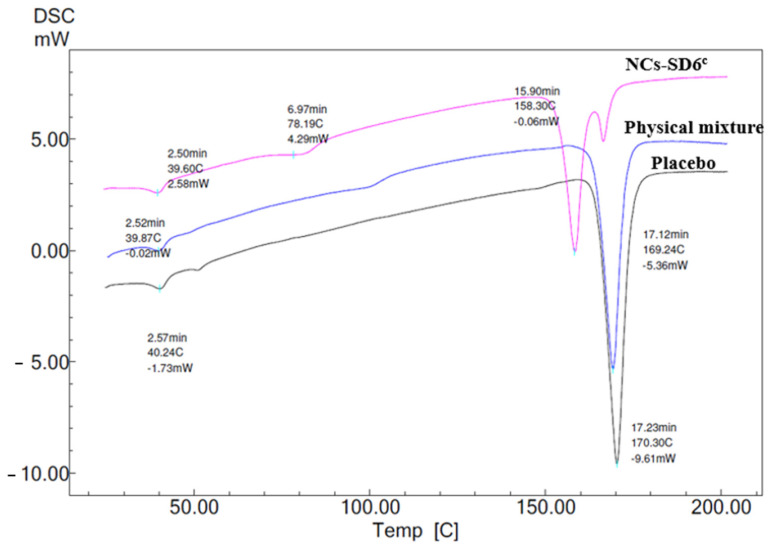
DSC curves of placebo, physical mixture, and spray-dried nanocrystals (NCs-SD6^c^).

The crystalline properties of spray-dried CFZ-NCs (NCs-SD6^c^) were evaluated by XRD, while solid-state thermal behavior was assessed using DSC analysis. In DSC analysis (Figure 5), endothermic peaks corresponding to P407 and D-mannitol were observed in the placebo and physical mixture samples at approximately 40 °C and 170 °C, respectively. In the physical mixture, the CFZ melting endothermic appeared at around 100 °C, whereas in the NCs-SD6^c^, this peak was shifted downward by approximately 20 °C. The reduction in the CFZ melting temperature following spray drying can be attributed to eutectic-like and plasticization effects arising from the intimate nanoscale mixing of CFZ with stabilizers (e.g., P407, HPMC) and the hydrophilic matrix of the former [54]. Such interactions are known to reduce lattice stability and increase molecular mobility at the crystal surface, leading to melting-point depression without a true polymorphic transformation, as widely reported for spray-dried drug nanocrystals [10,40]. In NCs-SD6^c^, an endothermic peak was observed at around 158 °C, followed by an exothermic event and then an endothermic peak around 170 °C. This thermal behavior can be attributed to the formation of the metastable δ form of mannitol (mp 150–158 °C), its subsequent crystallization to the α and/or β form, and the melting of the resulting crystal form [55]. Notably, it has been reported that even the metastable δ form of manntiol can remain stable for at least 5 years at room temperature (25 °C) when stored under dry conditions [56].

**Figure 6:**
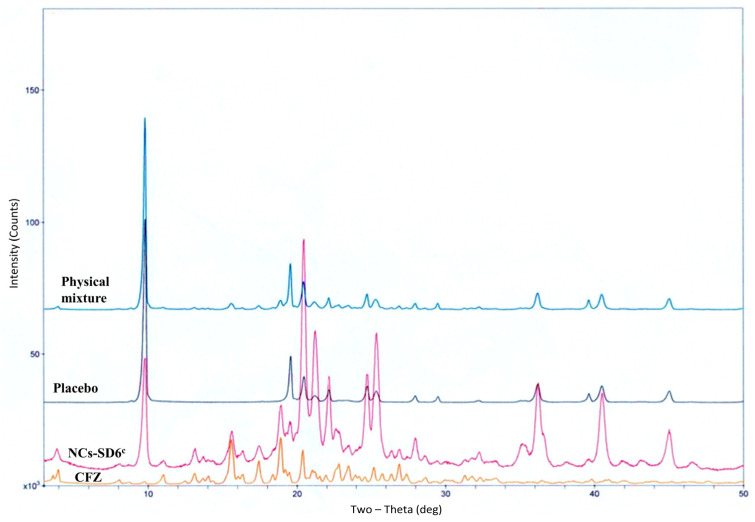
XRPD diffraction patterns of CFZ, placebo, physical mixture, and spray-dried nanocrystals (NCs-SD6^c^).

The diffractogram of dried CFZ-NCs (NCs-SD6^c^) (Figure 6) revealed the characteristic lines comparable to those of the pure CFZ sample, albeit with reduced intensity. Similarly to the fluidized bed granulation process, no evidence of polymorphic transformation was observed.

### 2.5. Evaluation of Micromeritic Properties

The micromeritic properties of the dried CFZ-NCs (i.e., NCs-FBG3 and NCs-SD6^c^) were investigated, and the results are given in Table 2. The dried CFZ-NCs produced by top spray granulation (NCs-FBG3) showed excellent (Grade 1) flow properties based on the Hausner ratio and compressibility index (%). According to USP <1174> (2016) [57], the angle of repose corresponded to Grade 3, indicating acceptable flowability. Overall, the obtained values indicate very good flow properties. These favorable properties can be attributed to the granulation process, which is widely employed in the pharmaceutical industry to overcome challenges associated with poor powder flowability [58,59]. In this study, CFZ-NS was utilized as the binder solution and sprayed onto the carrier material (lactose monohydrate) during granulation. Through this approach, nanometer-sized CFZ particles (205.70 ± 2.55 nm), together with the stabilizer agents present in the CFZ-NS, were uniformly incorporatedonto the carrier material particles, forming a homogeneous solid system. As a result, the CFZ-NS was successfully converted into a solid-state powder with high bulk density, good flowability, and improved tableting properties.

According to USP 46, the spray-dried CFZ-NCs (NCs-SD6^c^) showed “very, very poor” (Grade 7) flow properties based on Hausner ratio and compressibility index (%). In addition, the angle of repose could only be measured under conditions of agitation and vibration, further indicating poor flowability. During spray-drying, the nanosuspension was atomized into a hot air stream and dried within seconds as a result of intense heat and mass transfer. Consequently, the resulting powder properties were strongly affected by the drying conditions experienced by the droplets during the process, particularly with respect to bulk density, which was markedly reduced (0.23 ± 0.09 g/mL). This reduction in bulk density may be due to rapid solvent evaporation from the droplet surface, leading to the formation of a rigid outer shell. At elevated temperatures, water vapor generated within the particle can become trapped, resulting in increased internal pressure. The subsequent release of vapor through the particle pores may cause particle expansion and increased porosity, ultimately yielding a low-density powder [60].

The flow resistance and compressibility of powders are largely governed by interparticle interactions between small particles (typically < 10 μm) and larger particles, which arise from adhesive and cohesive forces acting at the particle surfaces. The most common interparticle forces include Van der Waals forces, capillary forces, and electrostatic interactions, and the magnitude of these forces plays a crucial role in determining powder behavior during mixing and processing [61]. Although top-spray granulation in a fluidized bed dryer offers advantages in terms of powder flow properties, it did not preserve the key benefits associated with nanocrystal technology in this study. This limitation was primarily attributed to time-dependent particle growth and aggregation phenomena following drying and redispersion, rather than a distinct crystal growth mechanism. In contrast, spray-drying was preferred for the conversion of the CFZ-NS to a solid form, as the primary objective was to minimize particle growth and aggregation of the dried CFZ-NCs. Consequently, the use of direct compression excipients was pursued to improve powder bulk density and, in turn enhance flow properties.

Silicified microcrystalline cellulose (Prosolv^®^ SMCC 90), a composite of microcrystalline cellulose (MCC) and colloidal silicon dioxide (CSD), was selected as a DC excipient to improve the bulk density and flow properties of the dried CFZ-NCs (NCs-SD6^c^). Silicification reduces powder bed stickiness and provides superior flowability compared with MCC of similar particle size. Owing to its good mixing performance, partly attributed to rough surface morphology, SMCC has been widely used in both direct compaction and roller compaction processes [62]. Moreover, the approximately fivefold increase in surface area enhances the binding properties of MCC, making SMCC particularly suitable for high-dose direct compression formulations. Consequently, the silicification process provides improved compressibility and tablet strength, enhanced flow properties, and reduced sensitivity to lubricants compared to MCC when used in direct compression manufacturing [63,64]. Following the incorporation of direct compression excipients into the dried CFZ-NCs (NCs-SD6^c^), excluding lubricants, their micromeritic properties were reassessed. The results demonstrated a substantial improvement in powder flowability and compressibility, rendering the formulation suitable for direct compression. Specifically, flow properties improved from “very, very poor” to “moderate” (Table 2). The observed increase in bulk density is primarily attributed to the use of SMCC as a filler, as its high surface area and binding capacity enabled it to occupy the gaps between large and small particles formed during spray-drying. Improved flowability and compressibility are directly related to tablet quality, particularly with respect to content uniformity, tensile strength, and tablet weight. Among these parameters, content uniformity and tablet weight are strongly influenced by die-filling consistency; otherwise, poor powder flow can lead to low die-filling and, consequently, increased variability in tablet weight and drug content [65].

The very poor flowability observed for the spray-dried CFZ-NCs can be attributed to their low bulk density, irregular particle morphology, and increased cohesiveness arising from their high surface area. Such characteristics are typical of spray-dried nanocrystal powders and commonly result in high interparticle friction and poor flow behavior. The incorporation of direct compression excipients, particularly silicified microcrystalline cellulose (SMCC), effectively compensated for these limitations by increasing bulk density, reducing powder stickiness, and improving particle packing, flowability, and compressibility.

### 2.6. Preparation and Characterization of Dried CFZ-NCs Tablets

Following spray-drying of the nanosuspension containing CFZ, stabilizers, and mannitol at twice the drug amount, a yield of 97.39% was obtained. Based on this yield, 371.828 mg of the spray-dried powder was equivalent to 100 mg of CFZ and was used for the preparation of NCs-SD-TAB by direct compression. Tablets were produced using an eccentric tablet press equipped with an oblong-shaped punch measuring 19.60 mm in length and 8.45 mm in width. The tablets exhibited a mean weight of 904.90 ± 2.19 mg, and a mean breaking force of 14.89 ± 0.61 kP. The disintegration time of the NCs-SD-TAB was determined as 12.35 ± 0.19 min, and the friability was calculated as 0.04 ± 0.00%, indicating adequate mechanical robustness. The drug content of the tablets (NCs-SD-TAB) was 99.53 ± 0.80%, which falls within the acceptable pharmacopoeial range of 95–105%.

**Figure 7:**
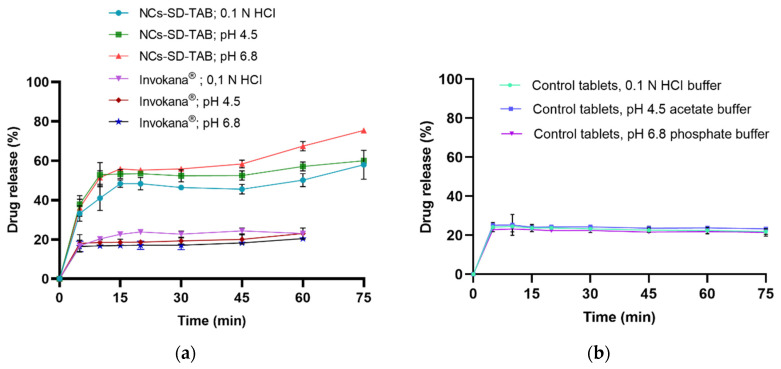
Comparative dissolution profiles of (NCs-SD-TAB), marketed product (Invokana^®^ 100 mg), (**a**) and control tablets (**b**) in different dissolution media.

The dissolution profiles of the marketed product (Invokana^®^ 100 mg) and the NCs-SD-TAB containing dried CFZ-NCs under non-sink conditions—namely 0.1 N HCI, pH 4.5 acetate and pH 6.8 phosphate buffer, which mimic gastrointestinal conditions—are presented in Figure 7a. For comparison, the dissolution profile of control tablet containing the same amount but unprocessed CFZ (d90: 18.1 µm) and formulated with the same tablet excipients as NCs-SD-TAB is shown in Figure 7b.

In 0.1 N HCI dissolution medium, the NCs-SD-TAB demonstrated an approximately 2.5-fold increase in CFZ release (58.01 ± 7.38%) compared with the marketed tablet product (23.64 ± 2.04%) and the control tablet (21.88 ± 1.40%) after 75 min. Similarly, in the pH 4.5 acetate buffer, the marketed product and the control tablet showed comparable drug-release values of 23.04 ± 2.79% and 23.31 ± 0.55%, respectively, while NCs-SD-TAB achieved a markedly higher release of 60.04 ± 0.95%, corresponding to an approximately 2.5-fold increase. In pH 6.8 phosphate buffer, the NCs-SD-TAB illustrated an even more pronounced enhancement, with about a 3.5-fold increase in drug release (75.41 ± 1.24%) compared with the marketed product (20.48 ± 1.48%) and the control tablet (21.45 ± 1.88%).

It is evident that CFZ, a BCS Class IV drug, exhibits poor aqueous solubility, resulting in a low dissolution rate in physiological media, which may contribute to variability and limited oral bioavailability. Notably, despite containing the same type and amount of stabilizers, NCs-SD-TAB demonstrated a significantly higher dissolution rate than the control tablets. This enhanced in vitro dissolution performance under non-sink conditions highlights the potential advantages of the nanocrystal technology. In detail, micro-sized CFZ (d90: 18.1 μm) was converted into redispersible nanocrystals (z-average: 219.8 nm), resulting in a substantial reduction in particle size and an increased surface-to-volume ratio. This reduction primarily led to a pronounced enhancement in the dissolution rate under gastrointestinal conditions. While a marked increase in saturation solubility is generally more prominent for particles below 100 nm, several studies have reported improved apparent solubility and/or oral bioavailability for drug nanocrystals in the submicron size range (≈200–300 nm), depending on the physicochemical properties of the drug and the formulation design [16,17,18,19]. Therefore, although enhanced in vitro dissolution does not universally translate into increased in vivo bioavailability, improved dissolution performance is widely recognized as a critical contributing factor that may support enhanced oral absorption in suitable cases [66,67,68,69,70].

### 2.7. Stability

The tablets (NCs-SD-TABs) were subjected to a three-month stability monitoring study under conditions of 25 ± 2 °C with 60 ± 5% RH and 40 ± 2 °C with 75 ± 5% RH. Physical quality control tests were performed at the end of the first and third months.

**Figure 8:**
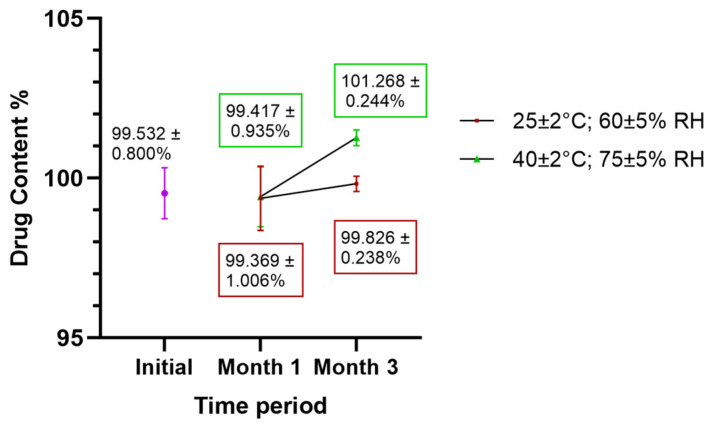
Drug content analysis of NCs-SD-TAB tablets during three months of storage.

The stability of the formulation was preliminarily assessed based on drug content analysis throughout the study period. The results demonstrated the CFZ content analysis throughout the study period. The results demonstrated that the CFZ content of the tablets remained within acceptable limits over the entire three-month period (Figure 8). No statistically significant differences were observed between the initial measurements and those obtained at the first and third months under either storage condition, at 25 °C (*p*-value = 0.951) or 40 °C (*p*-value = 0.716).

**Figure 9:**
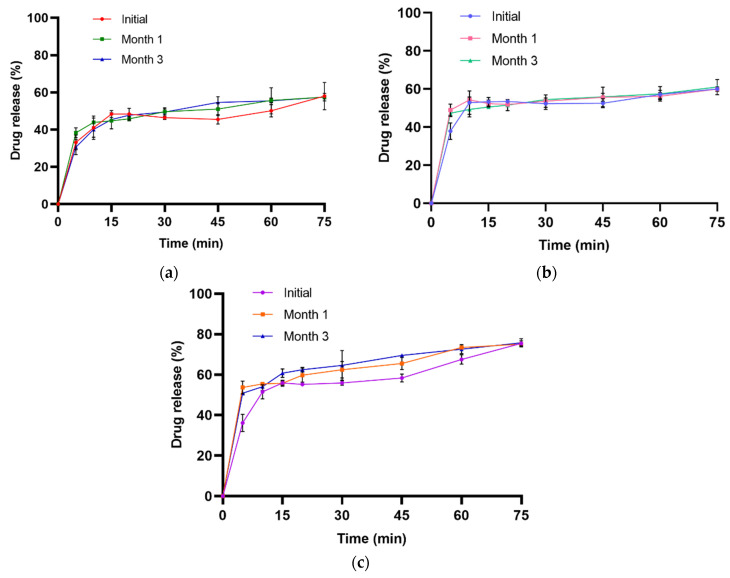
Dissolution profiles of NCs-SD-TAB in 0.1 N HCI (**a**), pH 4.5 acetate (**b**), and pH 6.8 phosphate (**c**) after storage at 25 ± 2 °C with 60 ± 5% RH.

Similarly, in vitro drug release studies were conducted in 0.1 N HCI, the pH 4.5 acetate buffer, and the pH 6.8 buffer for the tablets stored under both stability conditions for 3 months. NCs-SD-TAB stored at 25 ± 2 °C with 60 ± 5% RH exhibited a cumulative drug release of 57.43 ± 0.47% after one month and 57.41 ± 2.06% after three months in the 0.1 N HCl dissolution medium (Figure 9a). In the pH 4.5 buffer, the corresponding drug release values at the first and third months were 59.95 ± 1.21% and 61.03 ± 2.43%, respectively (Figure 9b), while in the pH 6.8 buffer, the values were 75.05 ± 0.75% and 75.77 ± 2.29%, respectively (Figure 9c). The results of the one-way ANOVA indicate that there is no statistically significant difference between the initial measurement and the measurements taken at the first and third months for the drug release in 0.1 N HCl (*p*-value = 0.978), pH 4.5 (*p*-value = 0.985), and pH 6.8 (*p*-value = 0.828).

**Figure 10:**
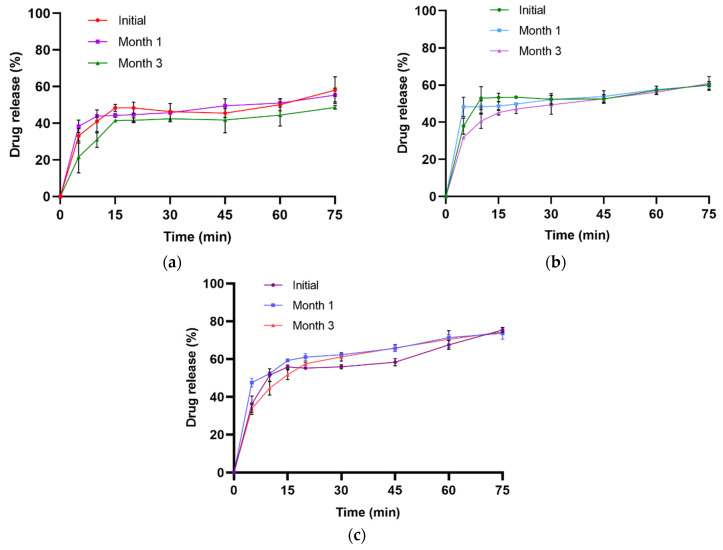
Dissolution profiles of NCs-SD-TAB in 0.1 N HCI (**a**), pH 4.5 acetate (**b**), and pH 6.8 phosphate (**c**) after storage at 40 ± 2 °C with 75 ± 5% RH.

Samples stored at 40 ± 2 °C with 75 ± 5% RH released 55.36 ± 1.78% and 48.63 ± 1.94% of the drug in 0.1 N HCl dissolution medium after the first and third months (Figure 10a). The drug release in pH 4.5 acetate buffer was 59.85 ± 2.05% at the first month and 60.88 ± 2.40% at the third month (Figure 10b). Similarly, release values of 73.68 ± 3.14% and 74.39 ± 0.88% were obtained in pH 6.8 phosphate buffer at the first and third months, respectively (Figure 10c). One-way ANOVA indicated no statistically significant differences between the initial measurement and those at the first and third months for drug release in 0.1 N HCl (*p*-value = 0.619), pH 4.5 acetate buffer (*p*-value = 0.871), and pH 6.8 phosphate buffer (*p*-value = 0.910).

It should be noted that drugs that undergo transformation of their crystal structure into an amorphous or metastable form following formulation or drying should be monitored during storage using techniques such as XRD and DSC, given the potential for conversion into a more stable crystal form [71]. In this study, DSC and XRD analyses were not included within the scope of stability testing, as CFZ retained its stable crystal structure following both nanomilling and drying processes. However, polymorphic transformation of an amorphous or metastable drug into a more stable crystalline form may result in reduced nanocrystal solubility and, consequently, diminished therapeutic efficacy [72]. Therefore, regulatory authorities increasingly require the identification, monitoring, and control of polymorphism at various stages of drug development and manufacturing [73].

The physical quality attributes of the tablets were also evaluated as part of the stability studies, and all results remained within the acceptable limits (see the Supplementary Materials, Figure S4). Based on these findings, tablets containing spray-dried CFZ-NCs and tableting excipients can remain stable over a storage period and under both tested conditions.

## 3. Materials and Methods

### 3.1. Materials

The purchase of canagliflozin (hemi-hydrate) (d90: 18.119 µm) was made from Fuxin Long Rui Pharmaceutical Co., Ltd. (Fuxin, China). D-Mannitol with different particle size grades (Pearlitol^®^ 50C, Pearlitol^®^ 160C) and alpha-lactose monohydrate grades (Granulac^®^ 200 and FlowLac^®^ 100) was supplied by Roquette, Geneva, IL, USA, and Meggle, Wasserburg am Inn, Germany, respectively. Magnesium stearate (Parteck^®^ LUB MST) and silicified microcrystalline cellulose (Prosolv^®^ SMCC 90) were obtained from Merck, Darmstadt, Germany, and JRS Pharma, Rosenberg, Germany, respectively. Acetonitrile (HPLC grade) and o-phosphoric acid (85%) were obtained from Merk Millipore, Darmstadt, Germany, and ISOLAB, Eschau, Germany, respectively, and were utilized as components of the mobile phase for the HPLC analysis.

### 3.2. Preparation of Optimized CFZ-NS

The CFZ-NS was optimized in our previous study [27]. Briefly, 1.30% hydroxymethyl propyl cellulose (HPMC-E15), 0.50% Poloxamer 407 (P407), 0.08% Tween 80 (T80), and 0.02% Sodium lauryl sulfate (SLS) were dissolved in deionized water, after which 4.25% CFZ was added. The resulting mixture was subjected to a wet milling process using a Dynomill^®^ (Willy A. Bachofen AG, Muttenz, Switzerland) for 1 h with 0.35 L zirconium beads (0.3 mm). HPMC is a hydrophilic polymer, while P407 and T80 are non-ionic amphiphilic surfactants that provide steric stabilization by forming a mechanical barrier between nanocrystalline drug particles. In contrast, SLS is an anionic surfactant that imparts an electrostatic charge to the particle surface. The combination of steric and electrostatic stabilisation mechanisms is commonly employed to obtain nanosuspensions and nanocrystals with enhanced physical stability and reduced particle size; this approach is referred to as electrosteric stabilization [27].

### 3.3. Solidification of CFZ-NS

The optimized CFZ-NS was subjected to fluidized bed granulation and spray-drying to convert it into a dry powder. The most appropriate drying method was subsequently selected based on characterization studies, with particular emphasis on achieving optimal redispersibility.

#### 3.3.1. Fluidized Bed Granulation

The granulation was carried out using a bench-top fluidized bed system (Mini-Glatt, Glatt Air, Ramsey, NJ, USA) equipped with the top spray configuration. Lactose and mannitol were selected as carrier materials due to their widespread use in pharmaceutical granulation and their contrasting physical properties, particularly in terms of hygroscopicity and crystallinity. Such differences are known to influence liquid retention, binder distribution, and granule formation behavior during fluidized bed processing. The use of both carriers therefore allowed an assessment of how carrier-related properties may affect granulation performance in nanocrystal-based systems. For each fluidized bed granulation process of NS (NCs-FBG), 100 g of carrier material was loaded into the product chamber, and 100 g of nanosuspension was sprayed onto the carrier as the binding liquid. The operational parameters applied are summarized in Table 3. Inlet air temperature ranges (55–75 °C) and air pressure settings were selected to ensure stable fluidization while minimizing the risk of premature drying or over-wetting of the carrier particles. The atomization pressure and pump speed were adjusted to facilitate controlled binder deposition and relatively homogeneous granule formation; however, some degree of agglomeration was observed under specific processing conditions. Outlet air temperature was continuously monitored as an indicator of process stability and drying efficiency.

#### 3.3.2. Spray-Drying

CFZ-NS was spray dried with or without the addition of mannitol using Buchi B-290 mini spray dryer (Flawil, Switzerland). Mannitol was incorporated at 1:1 and 1:2 ratios relative to the active ingredient concentration in nanosuspension. Spray-drying of CFZ-NS (NCs-SD) was performed to identify the optimal process parameters, as summarized in Table 4 (i.e., NCs-SD1^a^–NCs-SD6^c^). The inlet air temperature range (70–130 °C) and feed rate (10–30%) were selected to balance efficient solvent evaporation with the preservation of nanocrystal integrity. Initially, higher inlet temperatures and feed rates were evaluated to ensure complete drying. Subsequently, lower temperatures and reduced feed rates were applied to minimize thermal stress, excessive agglomeration, and irreversible particle growth. The spray air flow was adjusted to promote effective atomization and uniform droplet formation, thereby improving powder recovery and redispersibility. The aspiration rate was set to 100% to maximize the yield, as a high aspiration rate generates greater centrifugal force in the cyclone, facilitating particle collection. The nozzle assembly consisted of the nozzle tip with a 0.7 mm diameter hole and a 1.5 mm nozzle cap, which is typically used when compressed air serves as the atomizing gas.

### 3.4. HPLC Analysis of CFZ

The drug content of the nanosuspension, dried CFZ-NCs, and tablet dosage forms was quantified at 290 nm using a validated high-performance liquid chromatography (HPLC) method with a Waters Alliance 2695 system (Milford, MA, USA) equipped with 2996 photodiode array (PDA) detector, as previously described [27]. In addition, in vitro drug release studies were performed in dissolution media simulating gastrointestinal conditions (i.e., 0.1 N HCl, pH 4.5 acetate buffer, and pH 6.8 phosphate buffer) and were analyzed by HPLC under the same experimental conditions. The analytical method was also validated for each dissolution medium in terms of specificity, precision, linearity range, limit of detection (LOD), limit of quantification (LOQ), accuracy recovery, and stability.

### 3.5. Characterization of Dried CFZ-NCs

#### 3.5.1. Redispersibility Studies

The ability of dried NCs to redisperse upon contact with deionized water or biological fluids was evaluated by the redispersibility index. A predetermined amount of dried CFZ-NCs was accurately weighed and transferred to a 5 mL volumetric flask. Based on prior drug content analysis of the dried CFZ-NCs, the weighed amount was adjusted to correspond to the same drug concentration as that of the original nanosuspension used for particle size measurements. The volume was then made up with distilled water, and the suspension was subjected to ultrasonication for 3 min [74]. The mean particle size and particle size distribution were determined in triplicate using Litesizer 500 (Anton Paar, Graz, Austria) via dynamic light scattering.

A quantitative measure of the redispersibility of the sample was calculated according to Equation (2) [75], where D is the particle size of the redispersed nanocrystals and D_0_ denotes the particle size of the freshly prepared nanosuspension.

RDI% = D/D_0_ × 100 (2)

Redispersibility index values close to 100% are desirable, as they indicate that the nanosuspension can be completely redistributed to its original particle size after rehydration [40].

#### 3.5.2. Moisture Content

The moisture content of the dried CFZ-NCs was determined using an HR-83-P halogen moisture analyzer (Mettler Toledo, Melbourne, VIC, Australia) that operates based on the thermogravimetric principle. Moisture content was calculated by the instrument from the weight difference recorded before and after drying over a 5 min period. Each sample (approx. 2 g) was evenly distributed in the sample holder and heated to 105 °C, and the moisture content is expressed as a percentage.

#### 3.5.3. In Vitro Dissolution Test

In vitro drug release of CFZ from dried NCs was evaluated using USP Apparatus II Varian VK 7010 (Agilent Technologies, Santa Clara, CA, USA) under non-sink conditions in order to discriminate the behavior of the redispersed nanocrystals. A dissolution medium volume of 600 mL was selected based on the FDA Clinical Pharmacology and Biopharmaceutics Review of the marketed product, in which dissolution testing was conducted in 600 mL of water containing 0.75% SLS [10]. In the present study, SLS was intentionally excluded to assess the intrinsic dissolution behavior of the formulations under physiologically relevant pH conditions and to allow a more discriminative comparison between formulations. Dried CFZ-NCs samples corresponding to 100 mg of pure CFZ were carefully placed in 600 mL of dissolution medium, namely pH 4.5 acetate buffer and pH 6.8 phosphate buffer, maintained at 37 ± 0.5 °C. The paddle rotation speed was set at 75 rpm. Aliquots of 5 mL were withdrawn at 5, 10, 15, 20, 30, 45, 60, and 75 min; filtered through a 0.2 µm regenerated cellulose (RC) syringe filter (Sartorius AG, Göttingen, Germany); and analyzed using the validated analytical method described in Section 2.4. All experiments were carried out with three independently prepared tablets per formulation (n = 3) under identical conditions, and cumulative drug release is expressed as the mean ± standard deviation.

#### 3.5.4. Morphology and Solid-State Evaluation

The surface morphology of the unprocessed drug and the dried CFZ-NCs selected according to the higher redispersibility following the solidification process (i.e., NCs-FBG3 and NCs-SD6^c^) obtained using the two drying methods was examined by Scanning Electron Microscopy (SEM) using EVO 40 (Carl Zeiss AG, Cambridge, UK).

The thermal properties of the dried CFZ-NCs (i.e., NCs-FBG3 and NCs-SD6^c^) were analyzed by DSC (Mettler-Toledo, Greifensee, Switzerland). Samples were placed in aluminum pans and hermetically sealed under pressure. DSC measurements were carried out under a nitrogen gas over a temperature range of 25–200 °C with a heating and cooling rate of 10 °C/min.

XRPD analysis was carried out using an Ultima IV diffractomer (Rigaku, Tokyo, Japan) over a 2θ range of 3–50° at a scanning speed of 1°/min to examine the crystalline state of the dried CFZ-NCs. Furthermore, physical mixtures and placebo samples corresponding to each drying method were prepared to enable comparison of the thermal behavior and solid states of the dried CFZ-NCs. For the fluidized bed granulation technique, the physical mixture and placebo samples consisted of the nanosuspension formulation components (1.30% HPMC, 0.50% P407, 0.08% T80, and 0.02% SLS) with and without CFZ, respectively. Mannitol was also included in the physical mixture and placebo prepared for the spray-drying method, as it was used as a matrix former in this process.

#### 3.5.5. Micromeritic Properties

Hausner ratio and compressibility index: bulk and tapped densities were determined using a tapped density tester (SMV II Erweka GmbH, Langen, Germany) to calculate the Hausner ratio and compressibility index. A defined amount of dried CFZ-NCs (NCs-FBG3 and NCs-SD6^c^) was transferred into a graduated cylinder, and the initial bulk volume (V_0_) was recorded. The cylinder was then tapped 10, 500 and 1250 times, and the corresponding volumes (V_10_, V_500_ and V_1250_) were measured [76]. The bulk and tapped densities were employed to calculate the compressibility index (Equation (3)) and the Hausner ratio (Equation (4)) as follows, where ρt and ρb correspond to tapped and bulk densities, respectively.

Compressibility index (%) = ρt − ρb/ρt × 100(3)

Hausner ratio = ρt/ρb(4)

Angle of repose: Angle of repose was measured using a granulate flow tester (GTB, Erweka GmbH, Germany), following the method described by Sousa et al. (2023) [28]. An automatic laser optically measures the geometry of the powder cone, and the angle of repose is directly displayed by the instrument.

### 3.6. Tablet Formulations of Dried CFZ-NCs

After confirming the absence of major solid-state incompatibilities between CFZ and the tablet excipients by DSC analysis (Figure S5 in Supplementary Materials), tablets were prepared using unprocessed drug and spray-dried CFZ-NCs. The excipients listed in Table 3 were selected based on their well-established functionality in direct compression tablet formulations. SMCC was used as a multifunctional filler to improve flowability and compressibility, Ac-Di-Sol^®^ served as a superdisintegrant to promote rapid tablet disintegration, and magnesium stearate was included as a lubricant [77]. Spray-dried CFZ-NCs (NCs-SD6^c^), in amounts corresponding to 100 mg of CFZ, were blended with the selected excipients to prepare the NCs-SD-TAB formulation using the direct compression technique. Prior to mixing, Prosolv^®^ SMCC 90, Ac-Di-Sol^®^, and Mg stearate were passed through a 0.5 mm sieve. All excipients except Mg stearate, in the required proportions (Table 5), were blended for 10 min in a cube mixer (KB) connected to the drive unit via a Universal Gear (UG Erweka GmbH, Germany). Mg stearate was then added as a lubricant, and the mixture was blended for an additional 2 min. The final blend (NCs-SD-TAB) was compressed using a single-punch eccentric tablet press (Korcsh EK0 DMS, Berlin, Germany) with an oblong punch and die set measuring 19.60 mm in length and 8.45 mm in diameter. The tablet breaking force was maintained within the range of 12–15 kP. Control tablets containing unprocessed CFZ (d90: 18.1 µm) were prepared using the same excipients and processing conditions.

#### 3.6.1. Characterization Studies

Tablets containing CFZ-NCs (NCs-SD-TAB) were characterized in terms of weight variation, content uniformity, friability, tensile strength, disintegration time, and in vitro dissolution rate.

For weight variation analysis, 20 tablets were randomly selected and weighed individually. The mean tablet weight, standard deviation, and %RSD were calculated. Content uniformity was assessed according to USP guidelines [78]. Briefly, 10 randomly selected tablets were crushed using a mortar and pestle, and an amount of the resulting powder equivalent to the labeled dose was accurately weighed. Sample solutions were prepared at a 100% concentration, as described in our previous study [27], filtered through a 0.20 µm RC filter, transferred into vials, and analyzed using the validated quantification method. Tablet mechanical strength was assessed by determining friability and tablet breaking force. Friability was evaluated by placing 10 pre-weighed tablets in a friabilitor (Erweka GmbH, Germany) and rotating them at 25 rpm for 4 min following the USP standard procedure [79]. After completion of the test, adhering powder was removed from the tablets, and friability was calculated using Equation (5), where W_1_ and W_2_ refer to initial and final tablet weights, respectively).

Friability (%) = (W_1_ − W_2_)/W_1_ × 100 (5)

The disintegration time of the tablets was determined using a disintegration tester (DT2 Sotax, Westborough, MA, USA). Six tablets were placed in the tubes of the basket assembly and immersed in a beaker containing deionized water at 37 ± 0.5 °C, and the disintegration time was recorded [80]. The CFZ release behavior of tablets containing dried CFZ-NCs (NCs-SD-TAB), control tablets, and their marketed product (Invokana^®^ 100 mg) was investigated in 0.1 N HCl, pH 4.5 acetate buffer, and pH 6.8 buffer under non-sink conditions as a discriminative method, using the same procedure described in Section 3.5.3. 

#### 3.6.2. Stability Studies

The NCs-SD-TABs were transferred into high-density polyethylene (HDPE) bottles and stored in stability cabinets. The samples were monitored for 3 months under conditions of 25 ± 2 °C; 60 ± 5% RH and 40 ± 2 °C; 75 ± 5% RH. At the end of the first and third months, the tablet evaluation tests described in the preceding section were performed.

### 3.7. Statistical Analysis

Statistical analyses were performed using GraphPad Prism version 9.5.1 software (San Diego, CA, USA). Results are presented as mean ± standard deviation (SD), with a 95% confidence level and a significance threshold of *p* < 0.05. Comparisons between different groups were conducted using one-way analysis of variance (ANOVA) followed by Tukey’s multiple comparison test or an unpaired *t*-test, as appropriate. All experiments were performed in triplicate (n = 3).

## 4. Conclusions

This study provides a comprehensive comparison of top-spray fluidized bed granulation and spray-drying for converting CFZ-NS into nano-redispersible powders. Although both methods are commonly employed for drying nanosuspensions, particle size control was found to be more challenging during fluidized bed granulation. While reducing the feed rate or increasing the amount of carrier material may help mitigate agglomeration, such process adjustments must be carefully balanced against the drug load in the nanosuspension and the final weight of the nanocrystal-based solid dosage form. Spray-drying enabled the production of dried CFZ-NCs with high redispersibility (94%), and the resulting tablet formulation (NCs-SD-TAB) exhibited a 2.5-fold increase in in vitro drug release in 0.1 N HCl and pH 4.5 acetate buffer, as well as a 3.5-fold increase in pH 6.8 phosphate buffer, compared with the marketed product and control tablets. The enhanced dissolution performance of the nanocrystal formulation is associated with increased saturation drug solubility, which may facilitate improved adhesion to intestinal cell membranes and promote drug absorption. The improved dissolution behavior of NCs-SD-TAB under non-sink conditions, relative to the marketed and control tablets, is therefore expected to improve its oral bioavailability, potentially allowing dose and/or dosing frequency reduction and reducing the incidence of adverse events. Such improvements may ultimately contribute to better patient compliance and more effective diabetes management. Moreover, the use of industrially relevant and scalable drying techniques, particularly spray-drying, highlights the potential of the developed nanocrystal-based formulations for future scale-up and pharmaceutical manufacturing. Nevertheless, further permeability and in vivo studies are needed to fully elucidate the impact of nanocrystal formulation on intestinal transport and to confirm the potential improved oral bioavailability effect of the developed nanocrystal formulations of CFZ.

## Figures and Tables

**Table 1:** Particle size analysis of CFZ-NS and redispersed CFZ-NCs after drying and the redispersibility index of dried NCs.

Dried NCS Codes	Particle Size of CFZ-NS (nm ± SD)	Particle Size of Redispersed CFZ-NCs (nm ± SD)	Redispersibility Index (%)
**NCs-FBG1**	203.1 ± 2.70	360.2 ± 16.15	56.39
**NCs-FBG2**	203.1 ± 2.70	560.4 ± 44.28	36.24
**NCs-FBG3**	205.7 ± 2.55	291.8 ± 6.23	70.51
**NCs-FBG4**	205.7 ± 2.55	476.5 ± 17.55	43.17
**NCs-FBG5**	206.8 ± 1.24	389.8 ± 13.73	53.06
**NCs-SD1^a^**	205.0 ± 3.88	345.0 ± 9.61	59.41
**NCs-SD2^a^**	205.0 ± 3.88	420.6 ± 18.35	48.74
**NCs-SD3^b^**	206.7 ± 3.19	267.7 ± 3.43	77.22
**NCs-SD4^b^**	206.7 ± 3.19	272.9 ± 3.94	75.74
**NCs-SD5^b^**	206.7 ± 3.19	238.6 ± 1.96	86.63
**NCs-SD6^c^**	206.7 ± 3.19	219.8 ± 5.61	94.03

^a^: without mannitol. ^b^: mannitol:CFZ ratio is 1:1. ^c^: mannitol:CFZ ratio is 2:1.

**Table 2:** Comparative micromeritic properties of nanosuspension-derived powders obtained by fluidized bed granulation and spray drying, and powder blends used for NCs-SD-TAB tablet formulation.

Micromeritic Properties	Dried CFZ-NCs by Fluidized Bed Granulation (NCs-FBG3) (mean ± SD)	Dried CFZ-NCs by Spray Drying (NCs-SD6^c^) (mean ± SD)	Powder Blends of NCs-SD-TAB
**Bulk density (g/mL)**	0.46 ± 0.03	0.23 ± 0.09	0.87 ± 0.05
**Tapped density (g/mL)**	0.50 ± 0.03	0.37 ± 0.08	1.11 ± 0.04
**Hausner ratio**	1.10 ± 0.02	1.60 ± 0.04	1.28 ± 0.24
**Compressibility index (%)**	9.18 ± 0.75	39.84 ± 1.20	22.10 ± 0.58

**Table 3:** Process parameters of fluidized bed granulation and the type of carrier materials.

Parameters	Dried NCs-FBG				
	NCs-FBG1	NCs-FBG2	NCs-FBG3	NCs-FBG4	NCs-FBG5
**Inlet air temperature (°C)**	75–65	75–65	60–50	55	55–45
**Air pressure (bar)**	0.35–0.65	0.35–0.65	0.30–0.40	0.25–0.30	0.25–0.30
**Atomization (bar)**	1.0–1.25	1.0–1.25	1.0–1.25	1.0–1.25	1.0–1.25
**Pump speed (rpm)**	10	10	5	10	10
**Outlet air temperature (°C)**	28–24	28–24	25–23	25	25
**Carrier Material**	FlowLac^®^ 100	Pearlitol^®^ 160C	Granulac^®^ 200	Pearlitol^®^ 50C	Granulac^®^ 200

**Table 4:** Process parameters of spray-drying.

Parameters	Dried NCs-SD					
	NCs-SD1^a^	NCs-SD2^a^	NCs-SD3^b^	NCs-SD4^b^	NCs-SD5^b^	NCs-SD6^c^
**Inlet air temperature (°C)**	130	130	130	100	70	70
**Spray air flow**	30	30	30	50	50	50
**Feed rate (%)**	30	20	20	10	10	10
**Outlet air temperature (°C)**	44	52	52	56–55	41–42	40

^a^: without mannitol. ^b^: mannitol:CFZ ratio is 1:1. ^c^: mannitol:CFZ ratio is 2:1.

**Table 5:** The unit formula of NCs-SD-TAB containing dried NCs-SD6.

Components	Amount (%)
CFZ-NCs-SD^c^	41.314
Ac-Di-Sol^®^	3.8
Silicified microcrystalline cellulose (Prosolv^®^ SMCC 90)	54.386
Mg stearate	0.5

## Data Availability

The original contributions presented in this study are included in the article/Supplementary Material. Further inquiries can be directed to the corresponding author.

## Supplementary Materials

The following supporting information can be downloaded at https://www.mdpi.com/article/10.3390/ph19020240/s1, Figure S1: Particle size distribution of optimal CFZ-NS; Figure S2: Dissolution profiles of dried CFZ-NCs: (a) NCs-FBG1, (b) NCs-FBG2, (c) NCs-FBG4; Figure S3: DSC curve of API; Figure S4: The physical tests of tablet consisted of dried CFZ-NCS: (a) weight variation, (b) hardness, (c) disintegration time, (d) friability%; Figure S5: DSC curves of CFZ and tablet formulation (NCs-SD-TAB) components; Table S1: Moisture content (%) of dried CFZ-NCs by fluidized bed granulation (NCs-FBG) and spray-drying (NCs-SD) method.

## Abbreviations

The following abbreviations are used in this manuscript:

DM	Diabetes Mellitus
CFZ	Canagliflozin
SGLT-2	Sodium glucose co-transporter 2
CFZ-NS	CFZ-nanosuspension
CFZ-NCs	CFZ-nanocrystals
NCs-SD-TAB	Tablets containing dried CFZ-NCs
RH	Relative humidity
HbA1c	Glycated hemoglobin
BCS	Biopharmaceutical Classification System
NC	Nanocrystal
DC	Direct compression
NCs-FBG	Fluidized bed granulation process of NS
NCs-SD	Spray-drying of CFZ-NS
DSC	Differential Scanning Calorimetry
XRPD	X-ray powder diffractometry
Pe	Peclet number
