# Supplementary material for: Influence of Drying Methods on Redispersibility and Dissolution of Canagliflozin Nanocrystals: A Comparative Approach
Source: Pharmaceuticals (Basel). 2026 Jan 29;19(2):240. doi: 10.3390/ph19020240 (PMC12943712; doi:10.3390/ph19020240)
Supplement: Supplementary file 1 [file pharmaceuticals-19-00240-s001.zip › pharmaceuticals-4046378-supplementary.pdf]

pharmaceuticals

MDPI

Article

Supplementary Material: Influence of Drying Methods on Re-dispersibility and Dissolution of Canagliflozin Nanocrystals: A Comparative Approach

Yagmur Pirincci Tok, Burcu Demiralp, Sevgi Güngör, Ali Osman Sarikaya, Emre Erol Aldeniz, Udaya Kumar Dude, Yildiz Ozsoy

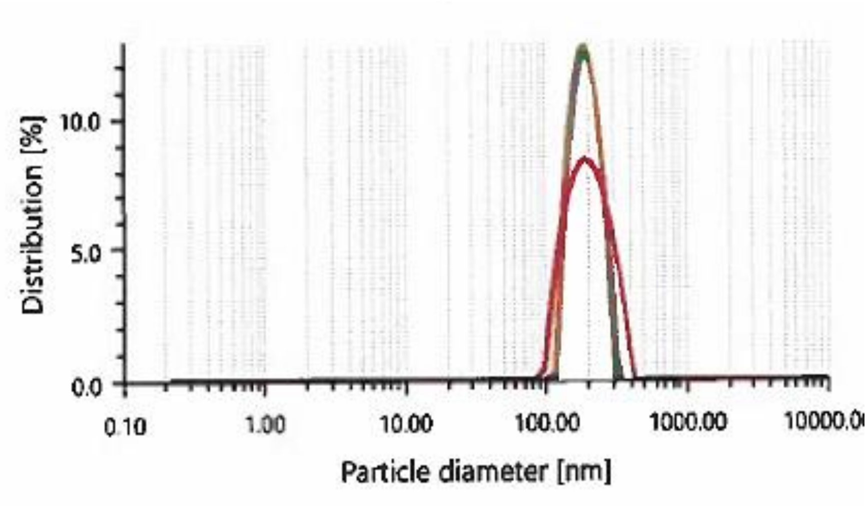

Figure S1. Particle size distribution of optimal CFZ- NS.

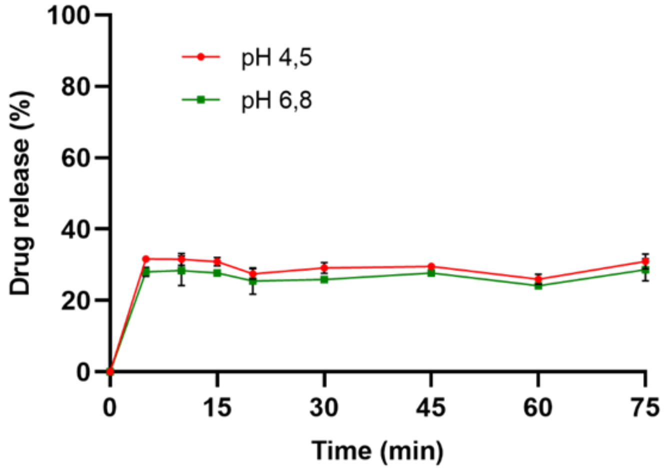

(a)

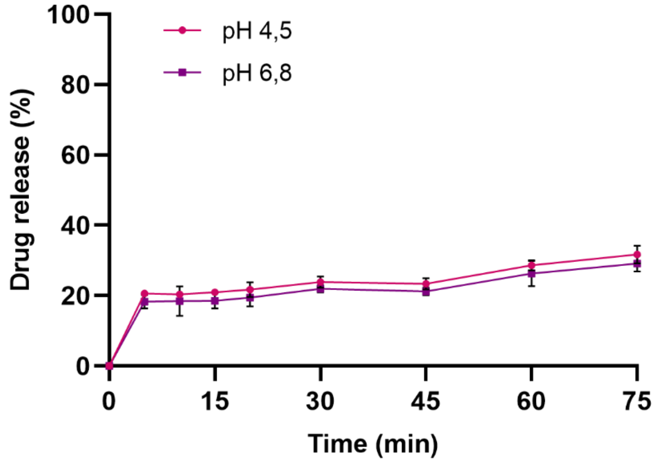

(**b**)

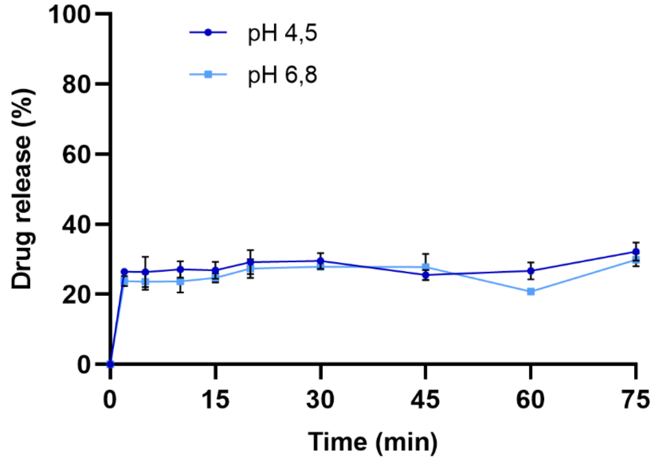

(**c**)

Figure S2. Dissolution profiles of dried CFZ-NCs; (**a**) NCs-FBG1, (**b**) NCs-FBG2, (**c**) NCs-FBG4.

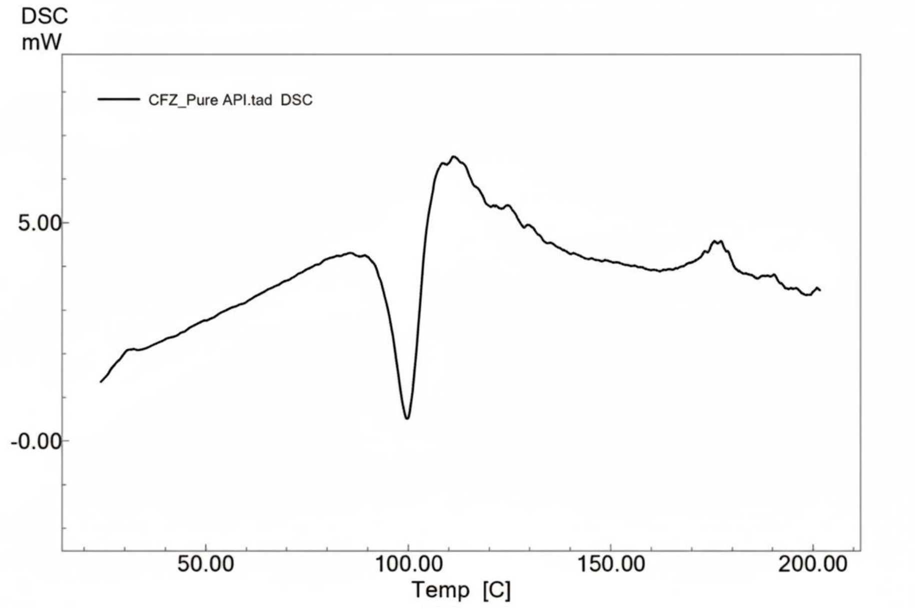

Figure S3. DSC curve of Canagliflozin.

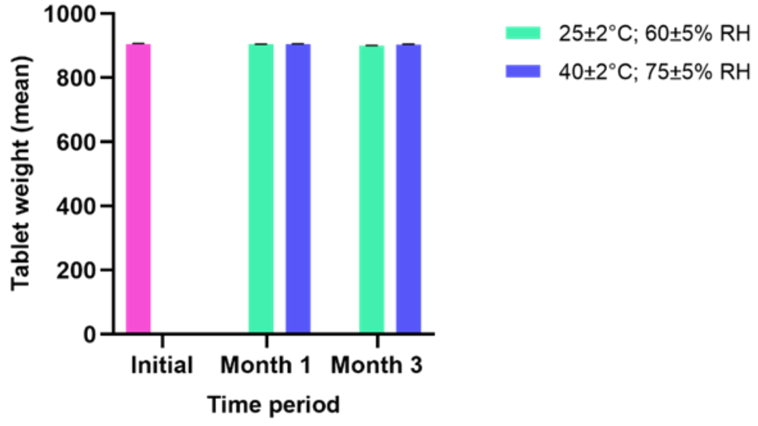

Tablet weight (mean)
1000
800
600
400
200
0
Initial
Month 1
Month 3
Time period
25±2°C; 60±5% RH
40±2°C; 75±5% RH

(a)

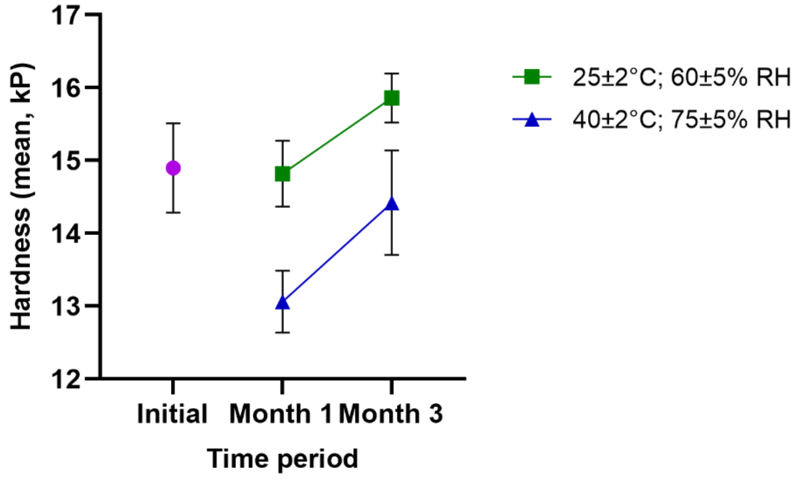

Hardness (mean, kP)
17
16
15
14
13
12
Initial
Month 1
Month 3
Time period
25±2°C; 60±5% RH
40±2°C; 75±5% RH

(b)

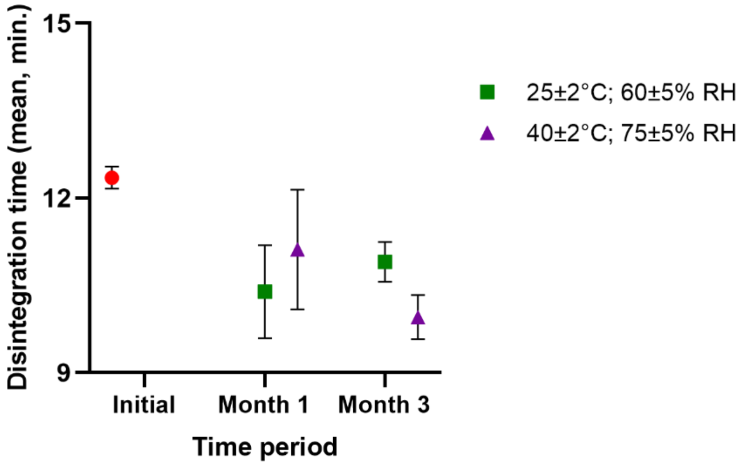

Disintegration time (mean, min.)
15
12
9
Initial
Month 1
Month 3
Time period
25±2°C; 60±5% RH
40±2°C; 75±5% RH

(c)

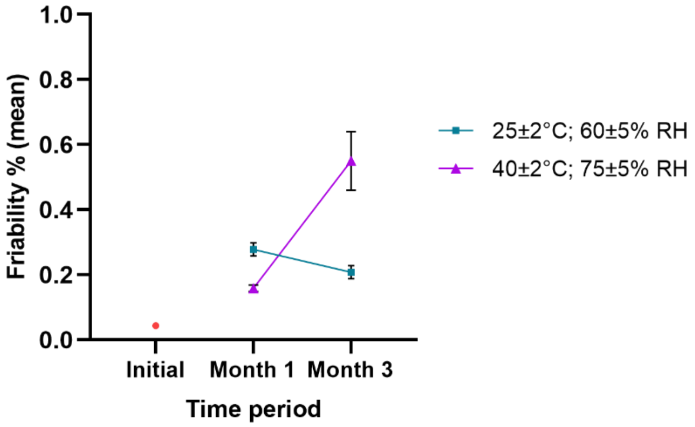

(d)

Figure S4. The physical quality tests of tablet containing spray- dried CFZ -NCS; (**a**) weight variation, (**b**) hardness, (**c**) disintegration time, (**d**) friability%.

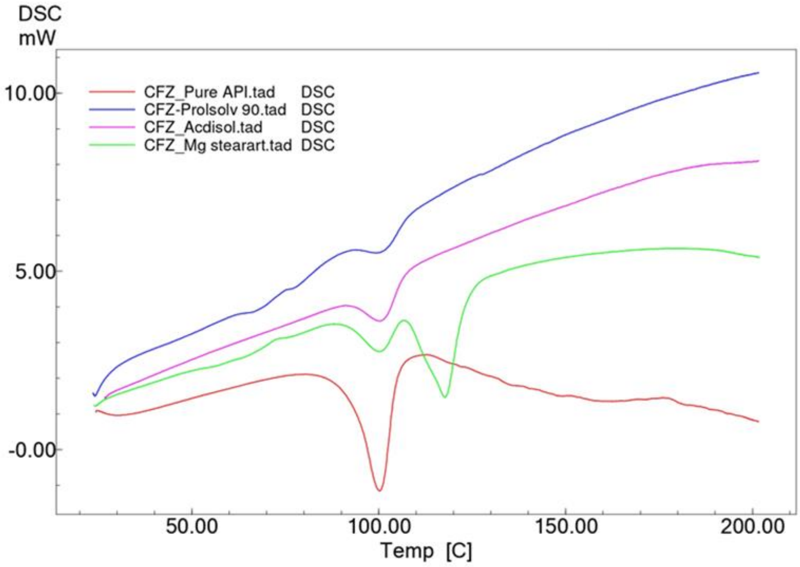

Figure S5. DSC curves of CFZ and tablet (NCs-SD-TAB) formulation components.

Table S1. Moisture content (%) of dried CFZ-NCs by fluid bed granulation (NCs-FBG) and spray drying (NCs-SD) method.

Dried CFZ-NCs	Moisture Content (%)
NCs-FBG1	0.79
NCs-FBG2	0.40
NCs-FBG3	0.89
NCs-FBG4	0.40
NCs-FBG5	1.72
NCs-SD1[a]	3.00
NCs-SD2[a]	2.10
NCs-SD3[b]	2.17
NCs-SD4[b]	6.31
NCs-SD5[b]	2.74
NCs-SD6[c]	3.17
